# Genetic assessment of three Fagaceae species in forest restoration trials

**DOI:** 10.7717/peerj.6958

**Published:** 2019-05-28

**Authors:** Patcharawadee Thongkumkoon, Siriwadee Chomdej, Jatupol Kampuansai, Waranee Pradit, Pimubon Waikham, Stephen Elliott, Sutthathorn Chairuangsri, Dia Panitnard Shannon, Prasit Wangpakapattanawong, Aizhong Liu

**Affiliations:** 1 Department of Biology, Faculty of Science, Chiang Mai University, Chiang Mai, Thailand; 2 Environmental Science Research Center, Faculty of Science, Chiang Mai University, Chiang Mai, Thailand; 3 Forest Restoration Research Unit, Department of Biology, Faculty of Science, Chiang Mai University, Chiang Mai, Thailand; 4 Key Laboratory for Forest Resource Conservation and Utilization in the Southwest Mountains of China (Ministry of Education), Southwest Forestry University, Kunming, China

**Keywords:** Chiang Mai, Framework tree species, Forest restoration, Genetic diversity, Microsatellite markers

## Abstract

Restoring isolated patches of forest ecosystems in degraded landscapes could potentially lead to genetic loss and inbreeding. Therefore, this study determined the occurrence of genetic diversity among the tree species *Castanopsis tribuloides*, *C. calathiformis*, and *Lithocarpus polystachyus* all of which were proven previously to be effective native tree species in the restoration of upland evergreen forests in northern Thailand when using the seed sample collection method. We tested our hypothesis as to whether the genetic diversity of a plant population that had been planted from the seeds of 4–6 adult trees would be lower and whether incidences of fixation index (Fis) would be higher among the second generation seedlings of these three Fagaceae species in isolated forest restoration trial plots. Microsatellite primers were selected from the entire genome sequence of *C*. *tribuloides* and the genetic sequences of *C. tribuloides*, *L. polystachyus*, and *C. calathiformis* were analyzed. Our results indicated a high degree of genetic diversity (He) in *C. tribuloides* (0.736) and *C*. *calathiformis* (0.481); however, a low level of genetic diversity was observed in *L*. *polystachyus* (0.281) within the restored forest. The fixation index for the second generation of *L*. *polystachyus* and *C*. *calathiformis* in the restored forest showed evidence of inbreeding. These results imply the efficiency of the seed sample collection method and verify that it does not reduce the level of genetic diversity in *C*. *tribuloides* and *C*. *calathiformis*. However, it may result in incidences of an inbreeding phenomena, suggesting the need to increase the number of adult trees used at the seed collection stage.

## Introduction

Deforestation in northern Thailand has resulted in numerous small, isolated forest fragments, within which plant inbreeding may occur, leading to levels of reduced genetic diversity and increasing the risk of species extirpations.

In northern Thailand, the Forest Restoration Research Unit of Chiang Mai University (FORRU-CMU) has adapted and modified the framework species method (Jalonen & Elliott, 2014) to restore evergreen forests in upland sites that have been degraded by agricultural practices (Elliott et al., 2006). Originally devised in North Queensland, Australia (Goosem & Tucker, 1995), the method involves planting 20–30 native tree species that have high survival and growth rates, broad dense crowns that suppress herbaceous weed growth, attract seed-dispersing wildlife, and are fire-resilient. This so-called framework tree species method is recommended for restoring northern Thailand’s upland evergreen forests, in which the following trees are found; Moraceae (e.g., *Ficus* spp.), Fabaceae (e.g., *Albizia* spp. and *Erythina subumbrans*), and Fagaceae (e.g., *Castanopsis* spp. and *Quercus* spp.), as well as those of other indigenous families, such as Euphorbuaceae (e.g., *Bischofia javanica*), Meliaceae (e.g., *Melia toosendan*), and Rosaceae (e.g., *Prunus cerasoides*) (Elliott, Blakesley & Anusarnsunthorn, 1998; Elliott et al., 2006).

Forest restoration success can be measured both in terms of the number of plant species that re-establish (Thomas et al., 2014) and the degree of genetic diversity within those species among the forest community (Freitas, Lemos-Filho & Lovato, 2008; Li et al., 2008; Yue et al., 2016). The use of native species for forest restoration provides both environmental and livelihood benefits, and the long-term success of forest restoration depends on the use of native tree species and genetic considerations for restoration (Bozzano et al., 2014). Nevertheless, one of the problems with planting trees during forest restoration projects is the possibility of genetic isolation and inbreeding, which can lead to a loss in genetic diversity, failure to adapt to environmental change and ultimately infertility (Burgarella et al., 2007; Thomas et al., 2014).

Previous studies investigating the genetics of native species using microsatellite markers showed high levels of genetic diversity and low to medium levels of inbreeding incidences in such species as *C*. *acuminatissima* (Bl.) A. DC., *Quercus semiserrata* and *Prunus cerasoides* D. Don within Doi Suthep-Pui National Park (Blakesley et al., 2004; Pakkad et al., 2004; Pakkad, Ueno & Yoshimaru, 2008). Genetic information concerning native tree species can be extremely useful for the selection of trees to plant within forest restoration programs (Pakkad et al., 2004).

We tested the hypothesis that trees in forest restoration plots planted from the seeds that collected from four to six adult trees would be associated with reduced levels of genetic diversity and represent increases in incidences of inbreeding phenomena within the second generations of plant populations in the fragmented areas.

## Materials & Methods

### Study site

The study site comprised forest restoration trial plots, in which the framework species method (Jalonen & Elliott, 2014) of the forest restoration program was tested. The plots were located in an expansive area of degraded agricultural land that had been cleared of primary evergreen forests for the cultivation of cabbages, corn, carrots, and other crops approximately 40–60 years prior. The study site was located in the upper Mae Sa Valley, Chiang Mai Province in northern Thailand. Different plots were planted with 20–30 indigenous tree species, which was typical of evergreen forests in 1998, 2000, and 2006 (i.e.,  18°51′46.62″N, 98°50′58.81″E, 1,200–1,325 m elevation). The plot system was 1–2 km from a large remnant of old-growth forest, was partially degraded, and had been used previously for opium poppy cultivation about 40–80 years ago. Framework species saplings planted in the plots were grown with locally-collected seeds (except for *C. calathiformis*) from nearby small-scale, community tree nurseries. Full details of the study site and plot system were published previously (Elliott et al., 2003).

### Species selection

Since Fagaceae is a characteristic well-represented tree family found in upland evergreen forests in northern Thailand (22 species of *Quercus*, *Lithocarpus* and *Castanopsis*; Maxwell & Elliott, 2001), several species were trialed in FORRU-CMU’s plot system. For the current study, three closely related species were selected from the lists of tree species that were planted in plots established in 1998, 2000, and 2006: *Castanopsis calathiformis* (Skan) Rehder & EH Wilsin, *Castanopsis tribuloides* (Sm.) A. DC., and *Lithocarpus polystachyus* (Wall. ex A. DC) Rehder, respectively. The specimens were identified by Dr. Wattana Tanming, from the Queen Sirikit Botanic Garden in Thailand. *Castanopsis* spp. and *Lithocarpus* spp. are classified as monoecious trees. These are widely distributed throughout temperate and tropical forest ecosystems (Chockchaichamnankit, Phengklai & Anamthawat-Jonsson, 2008; Manos, Zhou & Cannon, 2001), are the dominant tree species found in late successional or climax forests, are normally found and considered to be native species in mixed and evergreen broad-leaved subtropical forests in Southeast Asia (Shi et al., 2011; Zhang et al., 2007). Their pollens is dispersed by wind and/or insects, and seeds are dispersed predominantly by gravity or small rodents.

The genetic basis for the *C*. *calathiformis* planting stock was adult trees planted in the restoration trial established in 1998. These trees were grown from seeds collected in Chiang Rai Province (180 km away from the study site). Regarding *C*. *tribuloides* and *L*. *polystachyus* trees in the restoration plating plots, these were saplings collected from 4 to 6 adult trees from FORRU’s Phenology trail at Doi Suthep-Pui National Park.

### Sample collection

To investigate whether genetic diversity was maintained in the restored community during forest restoration, we selected the three Fagaceae species *L*. *polystachyus* (31 trees and 35 seedling samples), *C*. *tribuloides* (28 trees and 29 seedling samples), and *C. calathiformis* (17 adult tree samples and 118 seedling samples) for testing purposes (Table 1). Table S1 shows information for each of the collected samples.

**Table 1:** Sample collections and genetic information for *L. polystachyus, C. tribuloides*, and *C. calathiformis* in the natural forest and restoration plots from Ban Mae Sa Mai, Mae Rim District, Chiang Mai.

**Species**	**Populations**	**Genetic information**			
		*N*	Ho	He	Fis
*L*. *polystachyus*	Natural forest	51	0.377	0.550	0.314
	• Adult tree	26	0.356	0.564	0.369
	• Seedling	25	0.398	0.537	0.258
	Restored area	15	0.171	0.281	0.432
	• Adult tree (Plots 1998 and 2006)	5	0.200	0.302	0.337
	• Seedling (Plot 2000)	10	0.142	0.261	0.457
*C*. *tribuloides*	Natural forest	39	0.691	0.731	0.056^*^
	• Adult tree	10	0.658	0.722	0.088
	• Seedling	29	0.724	0.739	0.025
	Restored area				
	• Adult tree (Plots 2000 and 2006)	18	0.824	0.776	−0.062
*C*. *calathiformis*	Restored area (Plots 1998.1–1998.3 and 2006)	135	0.331	0.481	0.332
	• Adult tree	17	0.356	0.416	0.144
	• Seedling	118	0.331	0.481	0.441

**Notes.**

Sample size (*N*), observed heterozygosity (Ho), expected heterozygosity (He), Fis (Fixation index).

* significant at *P* < 0.05.

**Figure 1:**
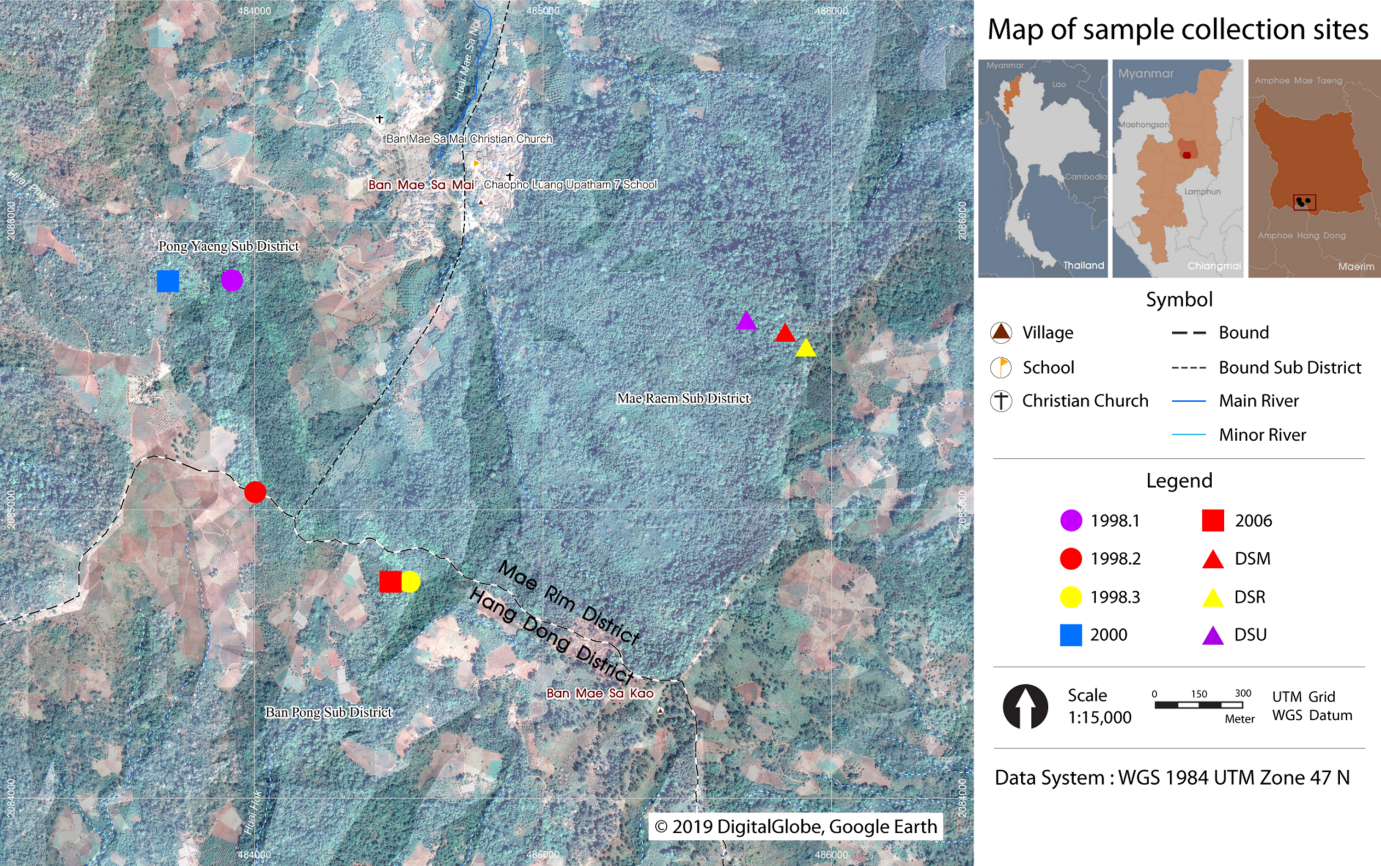
Map of sample collection sites. Three Fagaceae species were collected at Ban Mae Sa Mai, Mae Rim District, Chiang Mai, Thailand. DSR, Dong-Seng ridge line. DSU, Dong-Seng upper line. DSM, Dong-Seng middle line. Restoration plots planted in the year 1998 (1998.1–1998.3), 2000, and 2006. The figure was generated via the QGIS program (QGIS Development Team, 2014) and using the picture from Google Earth, DigitalGlobe, 2019.

Fresh leaf samples of the three Fagaceae species (trees and seedlings) were collected from restoration plots planted in the years 1998, 2000, 2006, and from the nearest natural forests, along the Dong-Seng ridge line (DSR), Dong-Seng upper line (DSU), and Dong-Seng middle line (DSM) (Jinto, 2009) (Fig. 1). Additionally, we collected young leaves for DNA extraction, which were dried with silica gel or directly frozen until use.

### DNA isolation and PCR amplification

Genomic DNA was extracted from leaves using a genomic DNA extraction kit (RBC^®^). The quality and quantity (Bassam, Caetano-Anollés & Gressholf, 1991) of the genomic DNA was quantified by NanoDrop^®^ and 1% agarose gel electrophoresis.

Nineteen primer pairs obtained from the entire genome sequence data of *C*. *tribuloides* were sequenced via Next Generation Sequencing (NGS) (Table 2) (Waikham et al., 2018). These primer sets were selected from the primers indicating polymorphism between individual samples of *C*. *tribuloides*. Twelve of the 19 primers were transferable for use with *C*. *calathiformis* and *L*. *polystachyus* (Table S2). Thus, the forward primers of the selected polymorphic loci were labeled with fluorescent dye(s): 6FAM, HEX and TAMRA (Shimizu et al., 2002). Then, the 12 microsatellite primers were used to amplify the individual samples of the three species. Amplification was carried out under the following conditions: initial denaturing at 95 °C for 4 min, followed by 35 cycles of 94 °C for 30 s, 57 °C for 30 s, and 72 °C for 30 s, with a final extension of 72 °C for 10 min. Notably, the annealing temperature ranged between 48 °C–64 °C for PCR amplification.

**Table 2:** Twelve microsatellite loci were developed for SSR markers and their amplification in the three species tested (Waikham et al., 2018).

**Primers**	**SSR motif**	**Primer sequence (5′–3′)**	**Amplification size range (bp**)		
			***L. polystachyus***	***C*.*tribuloides***	***C*.*calathiformis***
CT097	(ATT)_*n*_	F: HEX-CGACTTTGGGAAGGAAATAAAGG	140–152	139–175	126–144
		R: TGGACTTCAACTTGCCATAGTG			
CT110	(TGT)_*n*_	F: TAMRA-TTCTTCAGTTAGCCACATCG	172–184	169–205	173–179
		R: CGCTAAGTCCATACATACAACAG			
CT113	(TTC)_*n*_	F: HEX-CCACTCGTAGCAGCCAATAATA	114–195	144–171	115–175
		R: CCACTCGTAGCAGCCAATAATA			
CT127	(AGA)_*n*_	F: TAMRA-CCCAGAAAACGTATGATCTTTG	191–254	207–246	172–250
		R: CCATGCAACACTACCTCGTC			
CT128	(TCA)_*n*_	F: TAMRA-CCCTTGGCAGACAAACTAGATA	168–186	160–244	172–193
		R: GGCGCAACAACATATGAAGAAT			
CT132	(AT)_*n*_(AAG)_*n*_	F: TAMRA-TGACCCGAGCATGGTTTAT	158–203	126–174	178–208
		R: GGACGTTAGGCCTGTACATT			
CT135	(TGA)_*n*_(GAA)_*n*_	F: FAM-GCCTAGCTTATGGAGTGGTT	116–146	124–160	123–165
		R: GTCTTTGTGCAAGTGCTC			
CT149	(TCT)_*n*_	F: FAM-GCGCGTGACTTAGGCTCTTCAC	142–166	144–150	140–161
		R: CTTCTCTGTTGGCATTTCTTGC			
CT159	(TCT)_*n*_	F: FAM-ATCCATGTCCACTTCTTCAA	132–159	130–193	133–148
		R: CGTTTCCAAAACGAAGAAC			
CT161	(CAC)_*n*_	F: FAM-AACGATACTAGCGACCTTGA	145–166	136–166	139–166
		R: GCGAAAAACGCTCTCCAAC			
CT164	(CAC)_*n*_	F: HEX-ACAACACACCTAACATCACAAC	146–164	129–174	132–159
		R: GAATGTTGCTCAGCGAAG			
CT165	(CTT)_*n*_	F: HEX-AGCGCCTTCTTAATAGAACC	153–186	112–149	119–161
		R: TGGTGACCATTACTTGTTGA			

The PCR products were run on a 1.4% agarose gel to verify polymorphism. After which, PCR products sizes were determined by fragment analysis using a 96-capillary Applied Biosystems 3730xl DNA analyzer at the Germplasm Bank of Wild Species, Kunming Institute of Botany, Chinese Academy of Sciences, China. Allele size of each of the primers was sized using GeneMarker^®^, a genotyping software, version 2.6.4 (Soft Genetics LLC). The microsatellite allele sizes were sized by comparing the peak of each sample with references of allele sizes, which were then converted into alleles size and entered into a spreadsheet (Hulce et al., 2011).

### Data analysis

The microsatellite primers that were developed from the NGS data of *C*. *tribuloides* were applied for use with other related species, such as *C*. *calathiformis* and *L*. *polystachyus*, and were described with percentage of transferability. The transferability of the microsatellite primers was defined from the ratio of amplified/tested markers.

Genetic information concerning the populations of each of the species was estimated from the expected (He) and observed (Ho) heterozygosity using GenAlEx version 6.5 (Peakall & Smouse, 2012). The fixation index (Fis) was evaluated by POPGENE32 software version 1.32 (Yeh et al., 2000). The paternity of each seedling was determined by comparing adults with seedlings using the CERVUS software version 3.0.7 (Marshall et al., 1998) based on maximum likelihood paternity analysis. Parent pair analysis for unknown sexes was simulated using the defaulted parameter (80% and 95% of relaxed and strict confidence, respectively). The likelihood ratio was defined as a delta score representing how much more likely candidate parents and seedlings were to be related (Marshall et al., 1998).

To analyze the cryptic population structure, Bayesian cluster analysis in STRUCTURE 2.3.1 (Falush, Stephens & Pritchard, 2003) was used to assign the individuals into clusters based on their genotypes. Ten replicate STRUCTURE runs were performed, where the *K* value ranged from 1 to 10 and a burn-in and Chain Monte Carlo (MCMC) were presented as 100,000 replicates (Singh et al., 2013; Zong et al., 2015). To estimate the total population (*K*) in the samples, Structure Harvester was used (Earl & VonHoldt, 2012). The graphic developed by CLUMPAK (Kopelman et al., 2015) was used to illustrate the results.

## Results

### Acquisition and polymorphism of SSR markers

Nineteen primers selected from *C*. *tribuloides* were used for a PCR assay of 66 samples of *L*. *polystachyus*, 57 samples of *C*. *tribuloides*, and 135 samples of *C*. *calathiformis* (Table S2). Thirteen primers including CT097, CT110, CT113, CT127, CT128, CT132, CT135, CT149, CT154, CT159, CT161, CT164, and CT165, were used for amplification of *L. polystachyus* and 12 primers (the same aforementioned set except for CT154) of *C. calathiformis*. Consequently, those primers were selected to study the genetic diversity and population genetic structure of the three Fagaceae species.

Cross-species tests showed that 12 primers could successfully amplify *C*. *calathiformis*, and 13 primers for *L*. *polystachyus*, with 11 primers being perfect microsatellite types and two being compound microsatellite types. Thus, the transferability ratio (amplified/tested markers) from *C*. *tribuloides* to *C*. *calathiformis* was 63.16 and from *C*. *tribuloides* to *L*. *polystachyus* was 68.42.

### Genetic diversity

To investigate whether the genetic diversity of the framework tree species was maintained in restored plots we analyzed the population genetic diversity of *L*. *polystachyus*, *C*. *tribuloides*, and *C*. *calathiformis* as well as the potential changes in genetic diversity from the adult trees to their offspring.

For *L*. *polystachyus*, we also considered the samples collected from the restored forest as a single population and recorded its genetic diversity indexes, including observed heterozygosity (Ho) = 0.171, expected heterozygosity (He) = 0.281, and fixation index (Fis) = 0.432. We analyzed two subpopulations of adult trees and seedlings, resulting in the genetic indexes of 0.200, and 0.142 (Ho), and 0.302 and 0.261 (He), and 0.337 and 0.457 (Fis), respectively. In contrast, we also considered the samples collected from the nearest natural forest as a single population and the genetic diversity indexes were recorded as 0.377 (Ho), 0.550 (He) and 0.314 (Fis). While considering the two subpopulations of adult trees and seedlings in the natural forest, we found that the genetic diversity indexes of the subpopulations were 0.356 and 0.398 (Ho), and 0.564 and 0.537 (He), and 0.369 and 0.258 (Fis), respectively. These results suggest that the genetic diversity of the adult tree subpopulations versus seedlings might not differ in the restored areas. However, the subpopulation genetic diversity indexes of both the adult trees and seedlings collected from the restored areas were lower than those collected from the natural forest (Table 1).

For *C*. *tribuloides*, the samples were collected from the restored areas as a single population and the genetic diversity indexes were Ho = 0.824, He = 0.776 and Fis = −0.062. In the restored area, only *C*. *tribuloides* was found. In contrast, the genetic diversity indexes of the plant population taken from the nearest natural forest were 0.691 (Ho), 0.731 (He), and 0.056 (Fis). The genetic diversity indexes of the subpopulations for adult trees and seedlings were 0.658 and 0.724 (Ho), 0.722 and 0.739 (He), and 0.088 and 0.025 (Fis), respectively. This result showed that the genetic diversity of the trees that were planted in restored areas did not differ from that of the natural forest (Table 1).

For *C*. *calathiformis*, we collected samples from the restored areas. Notably, the population genetic diversity indexes were Ho = 0.331, He = 0.481, and Fis = 0.332 at the species level. The genetic diversity indexes of the adult tree subpopulations were Ho = 0.356, He = 0.416, and Fis = 0.144. The genetic diversity indexes of the seedling subpopulations were Ho = 0.331, He = 0.481, and Fis = 0.441 (Table 1).

Moreover, the paternity analysis of *L*. *polystachyus* was tested with CERVUS software by a parent pair (sex unknown) simulation with comparison of 31 candidate parents and 35 progenies. The success rate was reflected by a strict confidence level at 4%, relaxed confidence level at 12%, and delta scores at 3.12. The result did not show any assigned progeny to be related to the candidate parent. For *C*. *tribuloides*, with 10 candidate parents and 29 progeny trees, the success rate at strict confidence was 26%, relaxed confidence was 49%, and delta score was 9.76. The result showed that three candidate parents were assigned to 15 progenies (Table S3). For *C*. *calathiformis*, with 17 samples of candidate parents and 118 samples of progeny, the success rate showed with a strict confidence level at 20%, relaxed confidence level at 34%, and delta scores at 5.31. The result revealed that nine candidate parents were assigned to 83 progenies (Table S3).

### Population structure

**Figure 2:**
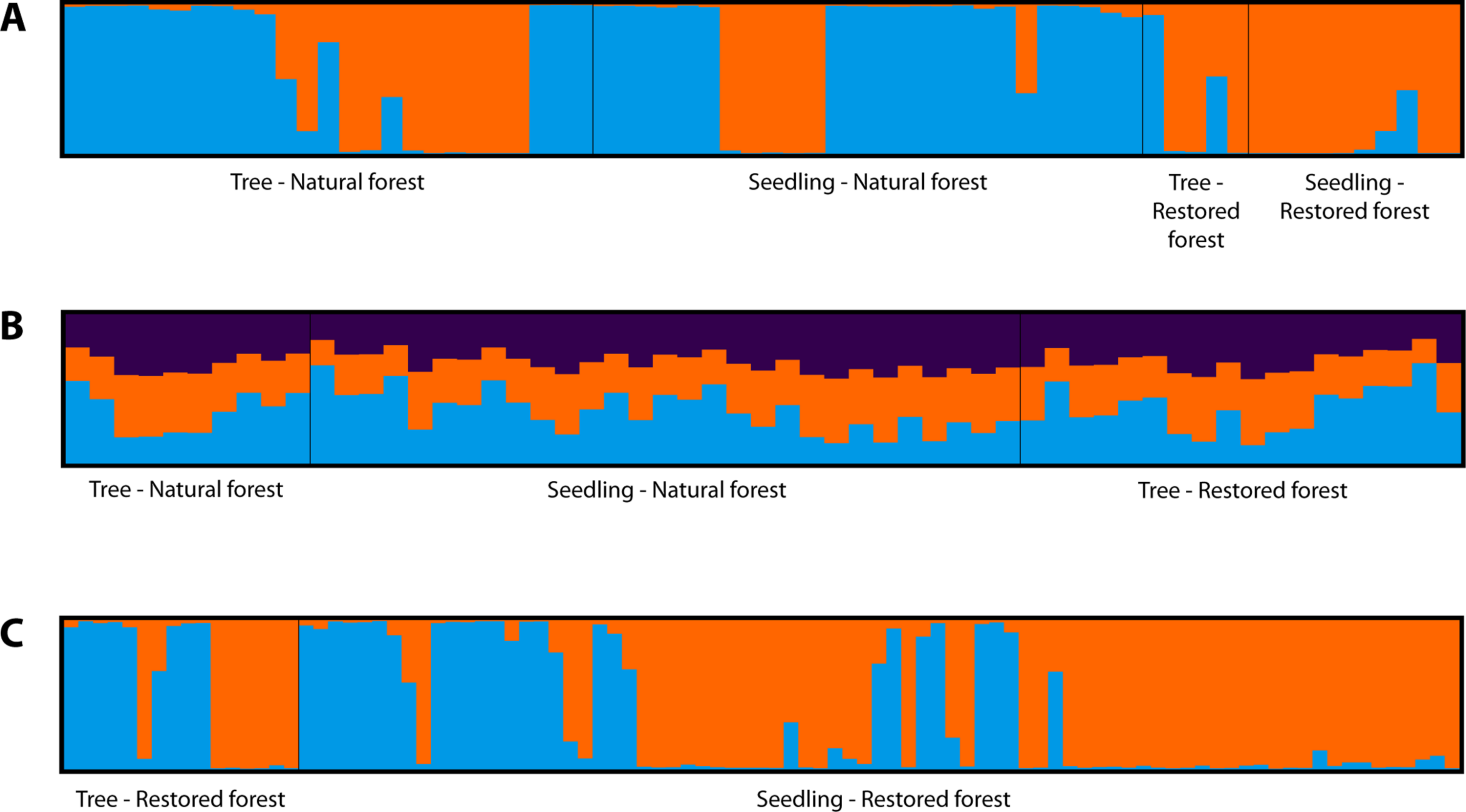
STRUCTURE analysis of three Fagaceae species in restoration areas and nearest natural forest. (A) *L. polystachyus* at *K* = 2. (B) *C. tribuloides* at *K* = 3. (C) *C. calathiformis* at *K* = 2.

We analyzed the cryptic population structure for each species based on Bayesian analysis via the STRUCTURE software. The Δ*K* reaches a maximum when *K* = 2, indicating that the most probable number of clusters was *K* = 2 for the non-admixture model of *L*. *polystachyus* and *C*. *calathiformis*. For *C*. *tribuloides*, the maximum Δ*K* was *K* = 3, thus the most probable number of clusters was *K* = 3 for the non-admixture model. As Fig. 2 shows, although we attempted to group samples into two clusters each for *L*. *polystachyus*, and *C*. *calathiformis*, and three clusters for *C. tribuloides*, all the populations were mixed together, and there was no distinct genetic structure found between subpopulations. These results indicate that there was no genetic differentiation occurring within the populations of each species.

## Discussion

Several Fagaceae species (including *Castanopsis* and *Lithocarpus*) were given priority during forest restoration in a previous project as they are known to be native and dominant species in late successional forests, and/or some species are pioneer species in south Asian forests (Huang et al., 2009; Shi et al., 2011). Based on the framework species method, >20 species in several plots of northern Thailand employed a mixed planting system for forest restoration over the last 20 years (Pakkad et al., 2002). Maintenance of the population of genetic diversity and achievement of a self-sustaining community is always a critical concern during forest restoration (Freitas, Lemos-Filho & Lovato, 2008). Thus, we selected three representative trees, *L*. *polystachyus*, *C*. *tribuloides*, and *C*. *calathiformis*, to determine whether population genetic diversity was maintained during offspring generation in restored forests.

This research represents the first study on population genetic diversity and structure of *L*. *polystachyus*, *C*. *tribuloides*, and *C*. *calathiformis* in northern Thailand using microsatellite markers developed using NGS data from *C*. *tribuloides*. The success of cross-transferability between species depended upon their close genetic relationship and haploid chromosome numbers (Chockchaichamnankit, Phengklai & Anamthawat-Jonsson, 2008; Fan et al., 2013; Singh et al., 2014). Additionally, the transferability property of microsatellite markers within a genus and between genera reduced the amount of time needed for developing SSR primers for new species (Ekué, Gailing & Finkeldey, 2009).

In this research, the first novel 12 microsatellite primers from *C*. *tribuloides* uncovered via NGS (Waikham et al., 2018) were successful in amplifying the other two Fagaceae species, as was revealed by the transferability ratio. In this study, the transferability ratios of *C*. *calathiformis* (63.16) and *L*. *polystachyus* (68.42) were higher than that of *C*. *cuspidata* to *C*. *chinensis* (30.8) (Huang et al., 2009). *Castanopsis* and *Lithocarpus* revealed the same number of diploid chromosomes (Chockchaichamnankit, Phengklai & Anamthawat-Jonsson, 2008), resulting in a high ratio of transferability success. Moreover, the primers developed from *C*. *tribuloides* included a large number showing medium to high levels of polymorphism. Notably, this could be employed for amplification across species. Thus, the primer set derived from *C*. *tribuloides* will be useful for studying genetic information in other related species found in the restoration areas of northern Thailand, such as *C*. *acuminatissima*, *C*. *argentea*, *C*. *diversifolia*, *L*. *elegans*, *L*. *garrettianus*, *L*. *fenestratus*, *Quercus kerrii*, *Q*. *semiserrata*, and *Q*. *vestita* (Elliott et al., 2006).

The high levels of genetic diversity within the three Fagaceae species populations may be related to their biological characteristics, including being monoecious and outcrossing plants with high crossing rates as well as utilizing wind pollination while having a long life span (Blakesley et al., 2004; Nakanishi et al., 2015; Provan et al., 2008). Species with these characteristics are expected to show high levels of genetic variation (Hamrick & Godt, 1996). Moreover, genetic diversity in the plant species is associated with environmental heterogeneity (pollination and/or seed dispersal) and adaptation ability of the plant populations to respond to environmental changes (Gram & Sork, 2001; Kirk & Freeland, 2011). Thus, the genetic diversity of *L*. *polystachyus*, *C*. *tribuloides*, and *C*. *calathiformis* in restoration planting plots were used as representative data for the distribution heterozygosity in genetic populations within the restoration areas at Ban Mae Sa Mai, Chiang Mai, Thailand.

Moreover, the fixation index (Fis) results of *L*. *polystachyus* (0.432) and *C*. *calathiformis* (0.332) in restored forests showed evidence of inbreeding*.* Such inbreeding within this study may have been caused by an excess of homozygosity (Charlesworth, 2003). The expected heterozygosity (He) values of *L*. *polystachyus* and *C*. *calathiformis* in the restored area(s) were higher than the observed heterozygosity (Ho), leading to a positive fixation index (Fis) (Pakkad et al., 2004). We assume that the Fis result indicates occurrence of inbreeding in the population (Chung & Chung, 2010; El-Sayed et al., 2017). The seed dispersal mechanism of *Castanopsis* and *Lithocarpus* is via gravity; thus, seeds remain near mother trees. Consequently, inbreeding depression would increase, and gene flow could not be exchanged through interpopulation trends (Feng, Wang & Gong, 2014). Thus, seed dispersal by gravity may lead to the accumulation of related individuals from the same parental trees, as a short distance could increase inbreeding (Hahn et al., 2012). Furthermore, the *L*. *polystachyus* samples in this study were germinated from six adult trees taken from the phenology trail at Doi Suthep-Pui National Park. For *C*. *calathiformis*, seedlings were planted in the 1998 in the plots collected from Chiang Rai. The genetic information for *L*. *polystachyus* and *C*. *calathiformis* in the restored forest showed that a smaller number of plant stock for the collected seeds was not suitable for maintaining genetic diversity in the small fragmented areas. For *C*. *tribuloides*, the tree populations in the restored area revealed high genetic diversity, implying that a suitable amount of plant stock was collected from the seeds. However, in this study, we did not find *C*. *tribuloides* seedling in the restored area. Thus, we were not able to conduct a paternity test for this species in the restored forest. Furthermore, we found plenty of *C*. *calathiformis* seedlings in the restoration planting plots, indicating the ability of this alien plant species to adapt to these areas. However, this could interrupt the growth of the other plant species in the restoration areas.

The paternity analysis of the three Fagaceae species showed that some candidate parents were assigned to progenies by comparing SSR genotype data. These species are monoecious; thus, male and female trees could not be distinguished. Pollination was revealed to involve both self- and cross-pollination. The model used to test paternity was with an unknown sex model; therefore, some candidate parents were assigned to progenies, and some were not assigned. Moreover, the similarity of genotype backgrounds in parents and a low number of microsatellite loci could be decreased as a result of assignment between candidate parents and progenies (Hadadinejad et al., 2011). Furthermore, the success rate of assigned offspring with the parent(s) was related to the number of markers used with a high polymorphic status (PIC). When decreasing the number of primers that showed low PIC, the success of paternity assignment is high (Satyanarayana, Babu & Jahageerdar, 2015).

The analyses of the population genetic structure confirmed further that there is no difference between the adult tree and seedling subpopulations in both restored and natural forests. Frequent genetic exchange among the populations has prevented genetic drift within plant populations (Slatkin, 1985; Yue et al., 2016). Moreover, gene flow within populations was affected by the mating system, mechanisms of seed dispersal and pollen movement, geographic distributions and stages of succession, colonization, adult densities as well as natural selection (Teixeira, Rodríguez-Echeverría & Nabais, 2014; Zhang et al., 2015; Zong et al., 2015). Therefore, outcrossing and wind pollinated tree species are expected to have high levels of gene flow between populations (Provan et al., 2008). Thus, long-distance dispersal in *Castanopsis* species might be caused by sexual outcrossing, allowing transfer of allele(s) among populations (Nakanishi et al., 2012; Savolainen & Pyhajarvi, 2007). In this study, we lacked access to the relevant pollinator data, distance of pollen flow, wind direction and velocity, and the reproductive physiology of each species. In future studies, these details should be considered for a greater understanding of gene flow between the fragmented areas. Moreover, in the future, study areas should be surveyed, and seedling samples of *L*. *polystachyus*, *C*. *tribuloides* and *C*. *calathiformis* in both of the restored and nearest natural forests should be collected to further investigate relevant genetic information (e.g., inbreeding coefficient, degree of genetic differentiation, and gene flow from the nearest natural forest to seedlings in the restored forest) of the second generation to complete the genetic diversity analysis for these species.

Deforestation and habitat fragmentation can lower genetic diversity. These situations cause reduction in genetic diversity, plant population size, allelic richness, polymorphism, and heterozygosity, which can increase incidences of inbreeding and ‘bottleneck’ events in populations. Moreover, fragmentation reduces gene flow and outcross breeding, resulting in low genetic diversity (Aoki et al., 2014; Gerwein & Kesseli, 2006; Pakkad, Ueno & Yoshimaru, 2008).

High genetic diversity among planted seedlings should be the one of the key plant survival strategies for natural selection with changing conditions. Thus, forest restoration should be done from the seeds of each species that were collected from a large number of trees (20–25 per population) to ensure that they have adequate genetic diversity to adapt to changing conditions (Thomas et al., 2014). However, at the time when seedlings were collected from the phenology trail at Doi Suthep-Pui National Park, only 4 and 6 *C*. *tribuloides* and *L*. *polystachyus* adult trees show flowering and fruiting, respectively, and were able to be collected. Such a low number of genetic sources could lead to low genetic diversity in later generations, and thus, this was taken into consideration when interpreting data. For *C*. *calathiformis*, the seedlings that were planted in the 1998 plots collected from Chiang Rai showed high value(s) in the restoration plots among both adult tree and seedling populations. This result concerning the genetic diversity of *C*. *calathiformis* (restored area), and *C*. *tribuloides* (both in restored and natural forests) showed high values, but low levels for *L*. *polystachyus* (restored forest). Notably, *C*. *calathiformis* and *L*. *polystachyus* in the restored forest displayed a positive value of fixation index (Fis). It is probable that a decrease in genetic information is relative to the narrow range of the genetic basis of the planting stock (Rogers & Montalvo, 2004). Thus, the FORRU-CMU staff should collect seeds from a large number of adult trees to decrease the inbreeding value of *C*. *calathiformis* and *L*. *polystachyus* within restoration populations. Maintaining the minimum level of intraspecific diversity to that of its progeny will, in turn, be viable and allow researchers to produce viable offspring with higher level of genetic diversity. Adaptive genetic diversity in the long term will occur from reduced inbreeding, decreasing genetic diversity reduction from genetic drift, and increasing a population’s ability to adapt to future site conditions. Currently, there is less available information on genetic diversity data from various native species, especially regarding the tropical tree species that could be playing an important role in the restoration of degraded tropical ecosystems (Bozzano et al., 2014).

## Conclusions

This study assessed the genetic diversity and population structure of *L*. *polystachyus*, *C*. *tribuloides*, and *C*. *calathiformis*, utilizing the framework tree species used for restoration of upland evergreen forests in degraded sites within northern Thailand. Novel SSR markers developed from NGS data for *C*. *tribuloides* were successfully used to derive genetic information and determine genetic variability among these three closely related species. High genetic variability and heterozygosity within *C*. *tribuloides* (both within restored and the nearest natural forests) showed that the seed collection method used by FORRU-CMU effectively conserved genetic diversity during the forest restoration process. However, regarding *L*. *polystachyus* and *C*. *calathiformis* in the restored forest, an increasing Fis value was detected. Consequently, seeds for planting stock production should be collected from a larger number of adult trees to increase heterozygosity and reduce incidences of inbreeding. The genetic information from this study can be used for better planning of forest restoration and improved forest ecosystem conservation in the future.

##  Supplemental Information

10.7717/peerj.6958/supp-1 Table S1 Information for *L*. *polystachyus*, *C*. *tribuloides*, and *C*. *calathiformis* collected from sampling sites

10.7717/peerj.6958/supp-2 Table S2 Genetic information of 12 microsatellite primers for *L*. *polystachyus*, *C*. *tribuloides*, and *C*. *calathiformis* An average number of alleles (Na), Observed heterozygosity (Ho), Expected heterozygosity (He).
(-) no amplification
(*) amplified success in all taxa

10.7717/peerj.6958/supp-3 Table S3 Assignment of progeny with candidate parents

## Additional Information and Declarations

### Data Availability

The following information was supplied regarding data availability: The raw measurements are available in the Supplemental Tables. Table S1 showed the raw data information and the code of three Fagaceae species collected in this study. Table S2 showed genetic information of each primer that tests with three species of Fagaceae. Table S3 showed the result of candidate parent assigned with progeny.
